# Preparation and Characterization of Thermoplastic Potato Starch/Halloysite Nano-Biocomposites: Effect of Plasticizer Nature and Nanoclay Content

**DOI:** 10.3390/polym10080808

**Published:** 2018-07-24

**Authors:** Jiawei Ren, Khanh Minh Dang, Eric Pollet, Luc Avérous

**Affiliations:** 1 Polymer Processing Laboratory, Key Laboratory for Preparation and Application of Ultrafine Materials of Ministry of Education, School of Material Science and Engineering, East China University of Science and Technology, Shanghai 200237, China; jiaweiren1991@gmail.com; 2 BioTeam/ICPEES-ECPM, UMR CNRS 7515, Université de Strasbourg, 25 rue Becquerel, 67087 Strasbourg CEDEX 2, France; minhkhanh238@gmail.com; 3 Department of Packaging and Materials Technology, Faculty of Agro-Industry, Kasetsart University, Bangkok 10900, Thailand

**Keywords:** halloysite nanoclay, plasticized starch, glycerol, sorbitol, microstructure, mechanical properties

## Abstract

Nano-biocomposites based on halloysite nanoclay and potato starch were elaborated by melt blending with different polyol plasticizers such as glycerol, sorbitol or a mixture of both. The effects of the type of plasticizer and clay content on potato starch/halloysite nano-biocomposites were studied. SEM analyses combined with ATR-FTIR results showed that a high content of sorbitol had a negative effect on the dispersion of the halloysite nanoclay in the starchy matrix. XRD results demonstrated that incorporation of halloysite nanoclay into glycerol-plasticized starch systems clearly led to the formation of a new crystalline structure. The addition of halloysite nanoclay improved the thermal stability and decreased the moisture absorption of the nano-biocomposites, whatever the type of plasticizer used. Halloysite addition led to more pronounced improvement in mechanical properties for glycerol plasticized system compared to nanocomposites based on sorbitol and glycerol/sorbitol systems with a 47% increase in tensile strength for glycerol-plasticized starch compared to 10.5% and 11% for sorbitol and glycerol/sorbitol systems, respectively. The use of a mixture of polyols was found to be a promising way to optimize the mechanical properties of these starch-based nanocomposites.

## 1. Introduction

In recent decades, biopolymers (e.g., polymers directly extracted from the biomass) such as polysaccharides, and more generally bio-based polymers from renewable resources have attracted a great deal of attention from researchers and industry because of the increasing awareness of environment protection and the lack of certain specific fractions extracted from fossil reserves [1,2]. Among the biopolymers, starch is commonly considered as a promising alternative to traditional non-renewable, non-biodegradable and fossil-based polymers due to its availability, renewability, biodegradability, and biocompatibility [3,4]. Native starch is a complex polysaccharide sourced from plants, composed of two types of *α*-glucans, linear amylose and highly-branched amylopectin. These biomacromolecules are organized into a highly complex semi-crystalline structure that results from the biosynthesis of the starch granules by the plant [5,6]. Neat starch exhibits high brittleness and poor mechanical properties. However, the addition of plasticizers can improve the material flexibility and processability. It is now very well documented that with plasticizers and under thermomechanical input, the highly organized native starch can be disrupted and destructured into a molten continuous amorphous phase to obtain thermoplastic starch (TPS). Two types of plasticizers are usually used and combined with starch: a volatile plasticizer, mainly water, which also acts as a destructuring agent, and a non-volatile plasticizer such as polyols (sorbitol, glycerol) [7]. TPS shows great potential for short term applications e.g., agricultural mulch films and packaging [7,8,9,10]. Nevertheless, TPS still exhibits multiple shortcomings that limit its usage, such as weak mechanical properties compared to conventional thermoplastics, long post-processing aging before stabilizing, and high water sensitivity [11,12]. Some of these issues can be addressed by developing multiphasic systems (blends, composites, etc.). Recently, the use of nanofillers and the elaboration of starch-based nano-biocomposites have been recognized as a powerful solution to overcome these weaknesses [9,13,14,15]. Due to their high surface areas, nanofillers bring increased mechanical properties, improved thermal resistance, and reduced gas permeability [16] while preserving the biodegradability and biocompatibility of the starchy matrix [17].

Up to now, researchers have focused on layered silicates, especially montmorillonites (MMT) [16]. However, improving the material properties of these silicates requires a great deal of exfoliation of the MMT platelets in the starch-based nano-biocomposites, which leads to increased fabrication cost [18]. Alternatively, halloysite nanoclay with its large aspect ratio, easy availability, high functionality, good biocompatibility, and high mechanical strength, has the potential to elaborate nanocomposites with promising performances [19,20,21,22,23,24]. This nanofiller is a multi-wall kaolinite nanotube with a theoretical unit cell formulation Al_2_Si_2_O_5_(OH)_4_·*n*H_2_O, with *n* from 0 to 2, for hydrated and dehydrated halloysite, respectively. The tubular structure of halloysite results from the wrapping of the constitutive 1:1 clay layers under specific geological conditions. Typically, halloysite ranges in length from 300 nm to 1500 nm, with an external diameter of 40–120 nm and internal diameter of 15–100 nm [25]. In contrast to other silicates such as kaolinite and MMT, most of the hydroxyl groups are located in the interior of halloysite tubes while siloxane groups are located on the external surface. Some silanols/aluminols are also present on the outer surface, mainly at the edges of the platelets. This unique feature gives halloysite a low surface energy, which in turn reduces the extent of filler-filler aggregation in the matrix compared to other nanofillers [26]. Consequently, halloysite nanoclay has been successfully used by some authors to enhance the properties of glycerol-plasticized starch matrix [27,28]. However, the behavior and efficiency of other types of polyol-based plasticizers on the thermal and mechanical properties of starch/halloysite nanocomposites prepared by the melt-blending method have not been systematically evaluated so far.

Thus, the aim of this work is to study the effect of the addition of halloysite into a starch-based matrix which is plasticized by glycerol, sorbitol or by a mixture of them, so as to reduce the water sensitivity and improve the thermal stability and mechanical strength. In particular, the effects of the type of plasticizer and nanoclay loading on the structure and properties of the obtained starch nano-biocomposites will be investigated using different characterization techniques such as scanning electron microscopy (SEM), attenuated total reflectance-Fourier transform infrared (ATR-FTIR), X-ray diffraction (XRD), thermogravimetric analysis (TGA), moisture content measurement and uniaxial tensile testing methods.

## 2. Materials and Methods

### 2.1. Materials

Potato starch was supplied by Roquette (Lestrem, France) (80% starch content, 19.5% moisture content, 0.05% proteins and 0.2% ash). The amylose and amylopectin contents were 20% and 80%, respectively. Glycerol was a 99.5% purity product (Thermo Fisher Scientific, Illkirch-Graffenstaden, France). Sorbitol was kindly supplied by Tereos (Origny-Sainte-Benoite, France) with 98% purity. Polysorb^®^, a glycerol/sorbitol mixture (59/41 by weight) was kindly supplied by Roquette (Lestrem, France). The halloysite nanoclay with diameter of 30–70 nm and length of 1–3 µm (CAS 1332-58-7) was purchased from Sigma-Aldrich (Lyon, France).

### 2.2. Preparation of Nanocomposites

The nanocomposites were obtained from a two-step process with the first being the elaboration of a dry-blend (powder) by the mixing of starch, water and polyol. Such dry-blend protocol allows the subsequent preparation of plasticized starch with high plasticizer content without the exudation phenomenon, mainly thanks to the strong interactions established between the polysaccharide chains and the polyols [29]. Then, the nano-biocomposites resulting from the dry-blend powder were elaborated by thermo-mechanical input with the addition of nanoclay.

#### 2.2.1. Preparation of Plasticized Starch Dry-Blends

First, the starch/plasticizer dry-blends were prepared. In this process, native potato starch was dried overnight at 70 °C in a ventilated oven to remove the free water (ca. 10 wt.% of the materials depending on the atmosphere relative humidity and temperature). Then, the dried starch powder was introduced into a Papenmeier turbo-mixer, and the plasticizer (glycerol, sorbitol, or the mixture) was slowly added under mixing until a homogenous mixture was obtained. The mixture was then dried at 170 °C in a ventilated oven for 40 min and occasionally stirred, and then the dry-blend was recovered. To obtain adequate moisture content, a pre-determined quantity of water was added to the dry-blend after cooling and mixed in the turbo-mixer, resulting in a formulation containing 54 wt.% potato starch, 23 wt.% plasticizer and 23 wt.% water. Finally, the powder was stored in a polyethylene bag in a refrigerator at 6 °C overnight prior to processing.

#### 2.2.2. Nano-Biocomposites Elaboration

To obtain nano-biocomposites, the plasticized starch powder was processed, with the addition of 3 wt.%, 5 wt.% and 7 wt.% of halloysite nanotubes compared to the weight of the dry-blend powder, in a counter-rotating internal batch mixer, Rheomix OS (Haake Thermo Fisher Scientific, Illkirch-Graffenstaden, France), at 70 °C for 20 min with rotor speed of 150 rpm. After melt mixing, the mixtures were then compression molded (Labtech Engineering Company, Muang, Thailand) at 110 °C, applying 18 MPa for 15 min. The obtained films (thickness approx. 1 mm) were then stored at 57% relative humidity at 23 °C for at least three weeks before characterization to obtain stabilized properties. The samples are designated as XY where X stands for the type of plasticizer (G for glycerol, S for sorbitol or P for the mixture of glycerol and sorbitol—Polysorb^®^) and Y for the weight percentage of halloysite nanoclay.

### 2.3. Characterization Techniques

#### 2.3.1. Scanning Electron Microscopy (SEM)

The fracture surfaces of the samples were observed with a VEGA3 LM scanning electron microscope (TESCAN, Brno, Czech Republic). The film samples were mounted on a stub using double-sided adhesive tape and coated with a thin layer of gold (10–20 nm). The images were obtained at an operating voltage of 5 kV and ×10,000 magnification.

#### 2.3.2. Attenuated Total Reflectance-Fourier Transform Infrared (ATR-FTIR)

IR spectra were recorded using a Thermo Scientific Nicolet iS10 FTIR spectrometer (Thermo Fisher Scientific, Illkirch-Graffenstaden, France) in attenuated total reflectance (ATR) mode. IR spectra of films were obtained for wavenumbers ranging from 4000 to 650 cm^−1^ by accumulation of 32 scans at a resolution of 4 cm^−1^.

#### 2.3.3. X-ray Diffraction (XRD)

X-ray diffraction analysis was carried out on a Bruker AXS D8 ADVANCE (Bruker, Wissembourg, France) using Cu-K*α* radiation (*λ* = 0.1542 nm) operating at 50 kV and 40 mA. The scanning region of the diffraction angle (2*θ*) was from 10° to 50° with a step size of 0.02°.

#### 2.3.4. Thermogravimetric Analysis (TGA)

The thermogravimetric analysis was performed on a TGA Q5000 machine (TA Instruments, New Castle, DE, USA). Sample masses ranging from 2 mg to 3 mg were heated from 35 °C to 600 °C at the rate of 10 °C/min under air atmosphere (flow rate of 25 mL/min).

#### 2.3.5. Density and Moisture Measurements

Samples films were cut into square shapes. Then, the weight, length, width and thickness of the film were measured, allowing the calculation of the material density. Experiments were done in triplicate. The water contents of samples were estimated using a MB45 moisture analyzer (Ohaus, Parsippany, NJ, USA) considering the weight loss measured after 1 h of drying at 105 °C.

#### 2.3.6. Mechanical Properties

The uniaxial tensile strength, elongation at break and Young’s modulus were determined using a Universal Testing Machine MTS 2M (MTS, Eden Prairie, MN, USA). The samples were cut into a dumbbell shape and the tests were performed at room temperature with a constant deformation rate of 10 mm/min and a distance grip separation of 4 cm. Five specimens of each formulation were tested and the average values were calculated. Samples were conditioned at ambient temperature with relative humidity of 52 ± 2% for at least 3 weeks prior to testing.

## 3. Results and Discussion

### 3.1. Morphological Characterization

**Figure 1:**
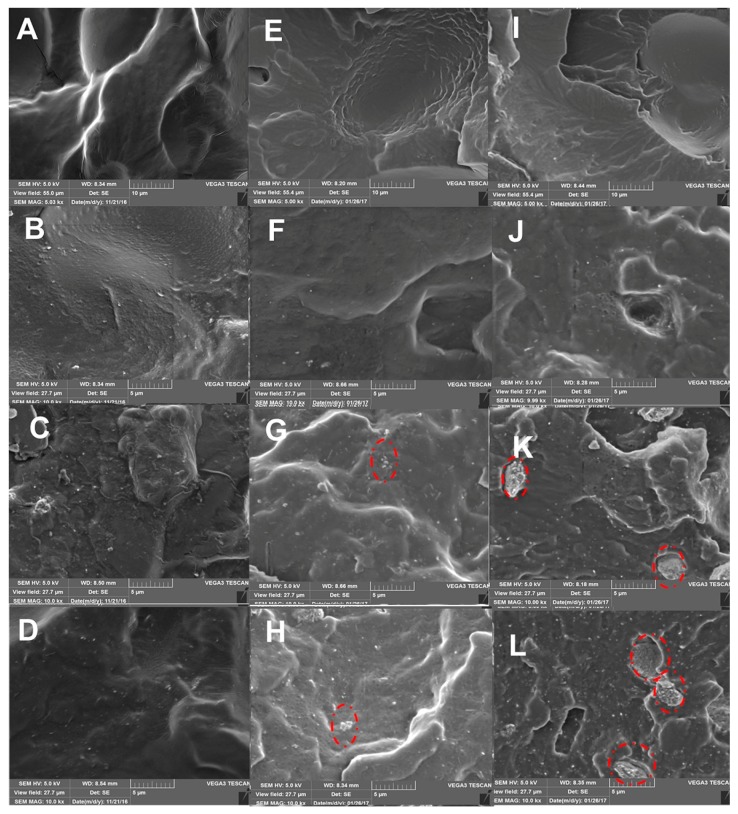
SEM images of the various plasticized starch systems and corresponding plasticized starch nano-biocomposites: (**A**) G0; (**B**) G3; (**C**) G5; (**D**) G7; (**E**) P0; (**F**) P3; (**G**) P5; (**H**) P7; (**I**) S0; (**J**) S3; (**K**) S5 and (**L**) S7. Scale bars are 5 or 10 microns.

SEM images of plasticized starch and starch nano-biocomposites are displayed in Figure 1. It can be observed that the control glycerol plasticized starch film (Figure 1A) exhibited uniform morphology without any obvious remaining starch granules, indicating that native starch structure was completely disrupted during the thermos-mechanical process. However, some voids were observed, resulting in discontinuous phases. By contrast, the addition of sorbitol or glycerol/sorbitol as non-volatile plasticizers (Figure 1E,I) resulted in more uniform morphologies and also no remaining starch granules. These observations could be explained by the greater shear force induced by sorbitol addition, leading to better plasticizer dispersion and continuous phases.

Adding halloysite into glycerol plasticized starch resulted in a more uniform and continuous morphology of the starchy matrix. No aggregates were visible for nano-biocomposites based on glycerol plasticized starch (Figure 1B–D). Even at the highest loading (7 wt.%) of halloysite (Figure 1D), homogenous clay dispersion with predominantly individual nanotubes was observed. The halloysite nanotubes were embedded into the matrix and no interfacial voiding was visible for glycerol plasticized samples, which implies a good interfacial adhesion between halloysite and the glycerol plasticized starch matrix (Figure 1C,D). However, large aggregates were clearly visible in the nanocomposites based on sorbitol plasticized starch at high loading (5 wt.% and 7 wt.%) of halloysite (red circles on Figure 1K,L).

Besides, the presence of voids between the clay aggregates and the starchy matrix suggested a poor interface quality (Figure 1K,L). For samples plasticized by the mixture of both polyols, halloysite nanotubes were also found to be uniformly dispersed in the matrix at the lowest content (3 wt.%) (Figure 1F). Limited amounts of very small aggregates and a large proportion of almost individually and randomly dispersed nanotubes were observed (Figure 1G,H) when the nanofiller content increased. The behavior of the nanocomposites based on starch plasticized by the mixture of glycerol and sorbitol was in-between those of the nanocomposites based on glycerol and sorbitol as plasticizers.

### 3.2. Chemical Interactions

**Figure 2:**
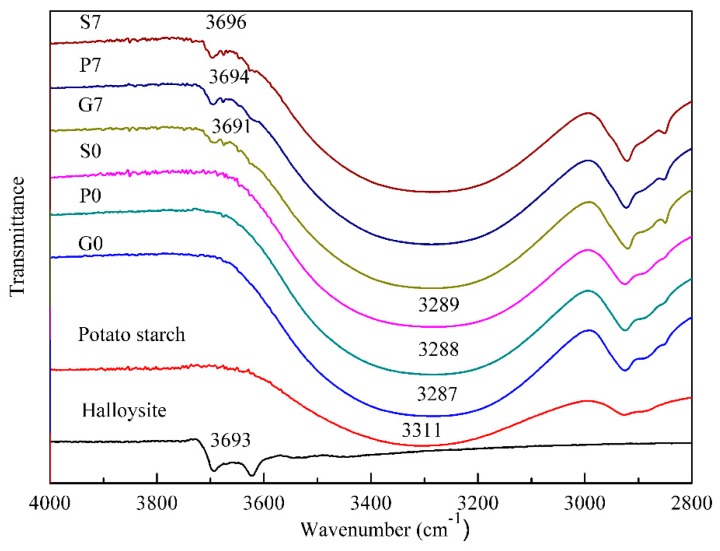
ATR-FTIR spectra of the native potato starch, halloysite nanoclay, unfilled plasticized starch matrices (G0, S0, P0) and plasticized starch nano-biocomposites containing 7 wt.% halloysite nanoclay (G7, S7, P7).

ATR-FTIR was used to investigate the interactions between plasticized starch and halloysite nanoclay. The spectra of the native potato starch, halloysite nanoclay and plasticized starch nano-biocomposites containing 7 wt.% halloysite nanoclay are shown in Figure 2.

The broad band between 3000 and 3600 cm^−1^ in the spectrum of native potato starch was attributed to the complex vibrational stretches associated with the free, inter- and intramolecular bound hydroxyl groups [30]. In the case of starch plasticized with glycerol, this band shifted to lower wavenumbers indicating strong and stable hydrogen bonds formation between the plasticizer and the starch macromolecules [31]. Tang, et al. reported similar behavior when incorporating nano-SiO_2_ into corn starch film [32] with the absorption band of O–H stretching shifted to lower wavenumbers, indicating an increase in intermolecular hydrogen bonds between nano-SiO_2_ and starch. The same trend was observed for the nanocomposites plasticized with sorbitol, alone or mixed with glycerol.

In the spectrum of halloysite nanoclay, the peaks at 3693 and 3623 cm^−1^ were related to the O–H stretching of inner-surface hydroxyl groups and inner hydroxyl groups, respectively. After addition into the glycerol plasticized matrix, the O–H stretching peak of inner-surface hydroxyl groups slightly shifted to a lower wavenumber, 3691 cm^−1^. Similar findings were previously observed by Schmitt et al. [27]. This shift could be attributed to the formation of interactions between the inner-surface hydroxyl groups of halloysite and the C–O–C groups of starch and/or glycerol. On the contrary, when halloysite nanotubes were added to sorbitol-plasticized matrices, the O–H stretching peak of inner-surface hydroxyl groups shifted to a higher wavenumber, 3694 and 3696 cm^−1^, respectively. This suggested a decrease in the intermolecular interactions between the inner-surface hydroxyl groups of halloysite and the C–O–C groups of starch and/or plasticizers. Since the main difference between these nano-biocomposites was the nature of the plasticizer, it could be concluded that glycerol formed stronger and more stable hydrogen bonds with the inner-surface hydroxyl groups of halloysite compared with sorbitol. This explains why a better dispersion of nanofillers could be obtained in the case of the glycerol plasticized starch matrix.

### 3.3. Microstructure and Crystallinity Studies

**Figure 3:**
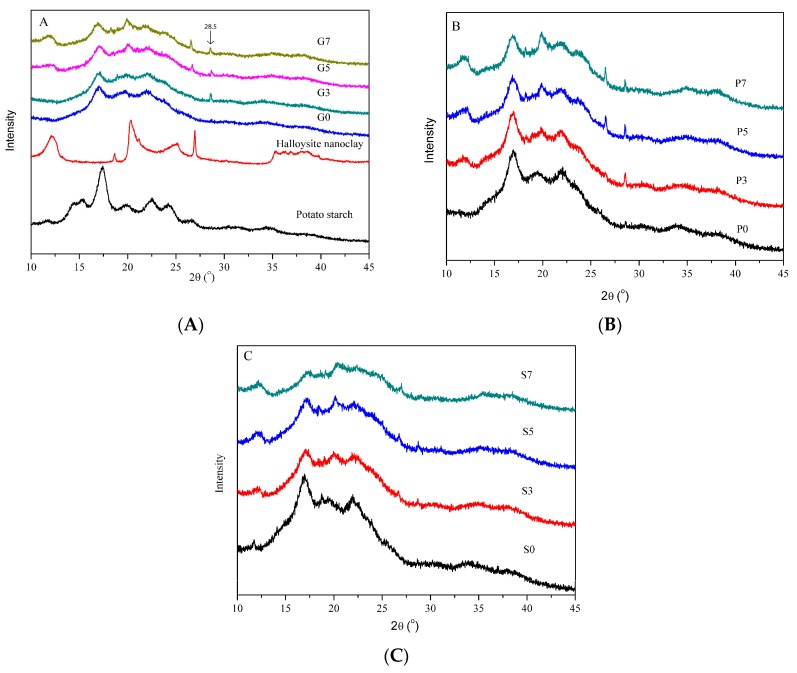
XRD patterns of the raw materials and the different nano-biocomposites with various nanofiller contents. From top to bottom, (**A**) native potato starch alone, halloysite nanoclay alone, and nano-biocomposites based on glycerol-plasticized starch; (**B**) nano-biocomposites based on glycerol/sorbitol mixture, and (**C**) nano-biocomposites based on sorbitol.

Figure 3 shows the XRD patterns recorded for native potato starch, the halloysite nanoclay and the plasticized starch/halloysite nano-biocomposites.

Halloysite displayed the typical pattern of the dehydrated form with characteristic peaks at 2*θ* of 12.1°, 18.6°, 20.3°, 25.0° and 27.0° [33]. The pattern of native potato starch showed diffraction peaks at 2*θ* of 15.4°, 17.4°, 19.9°, 22.5° and 24.2° which are characteristic of B-type starch crystalline structures [34]. After processing with glycerol, the B-type crystalline structure was transformed into E_H_-type and V_H_-type structures with characteristic peaks at 2*θ* of 17°, 19.9°, and 22.0°, respectively [35,36]. The E_H_-type structure is linked to amylopectin recrystallization while the V_H_-type structure is attributed to amylose crystallization into single helical structure [36]. Except for these three peaks, sorbitol plasticized starch showed weak and small diffraction peaks at around 12.0° and 18.8° which were assigned to conventional sorbitol crystallization during storage [37]. Importantly, matrices based on the glycerol/sorbitol mixture showed similar patterns to glycerol-based matrices indicating an absence of sorbitol recrystallization in such systems.

After dispersion in the starch matrix, the halloysite characteristic peaks at 2*θ* of 12.1° and 27.0° remained visible in the patterns of the nano-biocomposites. The peak intensity increased with the halloysite content while the peak position remained almost unchanged with only a slight broadening of the peak at 2*θ* of 12.1°. In addition, the characteristic peaks of plasticized starch were also observed in the diffractogram of the nano-biocomposites, attesting that halloysite nanotubes were well dispersed in the starch matrix [38]. No significant changes in the E_H_-type and V_H_-type crystallization peaks were observed for the starch/halloysite nano-biocomposites. The XRD patterns of nanocomposites based on glycerol displayed a new peak at 28.5°, compared with the pattern of unloaded plasticized matrix. Similar findings have been reported for starch/tunicin whiskers [39] and starch/sepiolite nanocomposites [40]. This was attributed to the formation of strong hydrogen bonds between the filler and the macromolecular system, facilitating amylopectin crystallization at the nanofiller interface. Thus, according to the ATR-FTIR analyses, it can be inferred that the interactions between the inner-surface hydroxyl groups of halloysite and the C–O–C groups of starch resulted in the appearance of a new crystalline structure. This phenomenon has been reported in the case of PP/halloysite [41], PA6/halloysite [42] and PVDF/halloysite nanocomposites [43]. However, the peak of the new crystalline structure was weak in the XRD patterns of sorbitol-based nanocomposites at high loading (5 wt.% and 7 wt.%) of halloysite. On the basis of SEM observation, this could be explained by the formation of halloysite aggregates in the sorbitol-plasticized matrix, decreasing their nucleating ability due to the smaller specific surface area, which hampered the amylopectin crystallization at the interface. Consequently, the new peak was more obvious in XRD patterns of nanocomposites based on the mixed sorbitol/glycerol compared to sorbitol–based nanocomposites due to the better dispersion state of halloysite nanoclays in the matrix.

### 3.4. Thermal Stability

**Figure 4:**
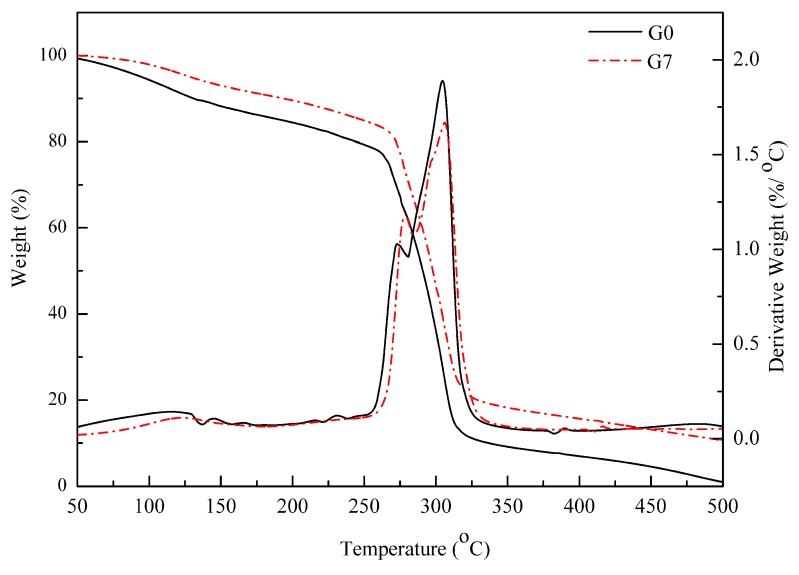
Weight loss (TG) and corresponding derivative (DTG) curves recorded for the unfilled glycerol-plasticized starch (G0) and the glycerol-plasticized starch nano-biocomposite containing 7 wt.% halloysite (G7).

Generally, the addition of nanoclays can generate two opposite effects on the thermal stability of polymer/clay nanocomposites: (i) a barrier effect which improves the thermal stability and (ii) a promoter effect which increases the thermal degradation process [44]. An enhancement in the material thermal stability is commonly observed in nanocomposite systems and is linked to the clay aspect ratio and dispersion state. The clay dispersion into the matrix increases the tortuosity of the combustion gas diffusion pathway and favors the formation of char at the material surface. Higher thermal stability can also result from strong interactions between the clay nanoparticles and the polymer matrix [45]. Also, the thermal degradation promoter effect is mainly due to the presence of hydroxyl groups on the edges of the clay which can catalyze the polymer degradation with a thermal dependence [46,47]. The barrier effect is predominant when the nanoclays are well dispersed in the matrix [47]. Thus, thermogravimetric analyses were performed on starch nano-biocomposites to analyze the impact of halloysite nanoclay on their thermal properties. Figure 4 presents the typical curves for glycerol plasticized starch and its nanocomposite with 7 wt.% halloysite. The curve of pristine plasticized starch shows three different degradation steps. The first step corresponds to the volatilization of water and glycerol, and the two other weight losses correspond to the degradation of amylose and amylopectin [48]. After incorporation of the nanoclay, the nanocomposite exhibits a further degradation at each step together with a slightly higher maximum degradation temperature, demonstrating an enhancement of thermal stability. Similar findings were also reported for starch/sepiolite nanocomposites [40].

The detailed TGA data for starch-based nanocomposites, and raw compounds are summarized in Table 1. The major parameters T_10%_, T_50%_ and T_90%_ refer to temperatures at which weight loss is 10%, 50% and 90%, respectively, and T_max_ corresponds to maximum degradation rate. The char residue (%) is the unburnt residue at 600 °C.

An increasing trend was observed for T_10%_ and correlated to the sorbitol content. This behavior was attributed to the higher thermal stability of sorbitol compared to glycerol. For all starch-based nanocomposites containing 3 wt.% halloysite nanoclay, T_50%_ was enhanced in comparison to the glycerol plasticized matrix, indicating that nanoclay had a barrier effect with an impact on degradation. This was attributed to the good dispersion of nanoclay in the matrix, even at low content. Considering the tube-like structure of halloysite nanoclay, an increase in the tortuosity was less obvious, thus the enhancement of thermal stability was more likely related to the strong interactions between the halloysite nanoclays and the matrix. Similar findings have been reported for polyethylene/halloysite [40], silicone rubber/halloysite [26] and polyurethane/halloysite nanocomposites [49].

T_50%_ gradually increased with halloysite nanoclay content in glycerol plasticized nanocomposites. However, there was no significant difference in T_50%_ as the nanoclay content increased from 3 wt.%, to 5 wt.% to 7 wt.% in nanocomposites of starch plasticized with sorbitol, alone or combined with glycerol. This finding suggests that some degradation promoter effect occurred and impacted the thermal stability with the addition of high levels of halloysite nanoclay. According to the SEM images, this could be related to the formation of aggregates in nanocomposites plasticized with sorbitol, alone or blended with glycerol, at high halloysite content (5 wt.% and 7 wt.%) which limited the barrier effect. Since the nanocomposites show different moisture content it is difficult to make a direct comparison of T_50%_ values, especially for such multiple-step degradation. Then T_max_, which corresponds to the temperature at maximum degradation rate was determined from the DTG curves maximum. In this case, the overall gradual increase in thermal stability with the clay content is more obvious and seems particularly marked for 7 wt.%. For T_90%_ and the char residue, whatever the type of plasticizer, both increased with the halloysite content due to the higher thermal stability of the halloysite nanoclays.

### 3.5. Density and Moisture Content

The densities and moisture content of the different samples were determined after three weeks of equilibration, at room temperature and 52 ± 2 %RH. Results are summarized in Table 2.

It can be seen that the nanocomposites showed a slightly increased density when nanofiller content increased. This is due to the higher density of halloysite nanoclay. Since the density of glycerol is lower than that of sorbitol, nanocomposites based on glycerol exhibited lower density than nanocomposites based on sorbitol. For the same reasons, the densities of the nanocomposites with the mixed polyols system were in-between those of glycerol and sorbitol-based plasticized starch nanocomposites.

Whatever the halloysite clay loading, the moisture content for nano-biocomposites with glycerol were higher than those recorded for nano-biocomposites with sorbitol alone or blended with glycerol. This is attributed to the higher hydrophilic character of glycerol compared to sorbitol [50]. Besides, the moisture content of nano-biocomposites showed an overall decrease with the increase in halloysite content. This was likely due to the relatively hydrophobic nature of the halloysite [25]. This last behavior could also be linked to the formation of interaction between the inner-surface hydroxyl groups of halloysite and the different groups of starch and plasticizers, thus decreasing the interaction between water molecules and polysaccharide chains and/or plasticizers molecules. The theoretical water contents of nanocomposites with the mixture of polyols were also calculated according to Equation (1):

W_mixture_ = W_glycerol_ V_glycerol_ + W_sorbitol_ V_sorbitol_ (1)

where W_glycerol_ is the water content of the nanocomposite with neat glycerol, W_sorbitol_ is the water content of the nanocomposite with neat sorbitol, V_glycerol_ and V_sorbitol_ are the respective nanocomposite volume fractions in the composites. The calculated values for sample P0, P3, P5 and P7 were 13.9%, 12.4%, 12.0% and 11.3% respectively. Interestingly, the experimental values were slightly lower but still in good agreement with the theoretical ones.

### 3.6. Uniaxial Tensile Properties

Young’s modulus, tensile strength and elongation at break of the different unfilled plasticized starch matrices and corresponding nano-biocomposites were measured. The main results are summarized in Table 2. It can be seen that the plasticized starch stiffness was greatly affected by the type of plasticizer. The sorbitol plasticized starch had the highest Young’s modulus while the glycerol plasticized starch showed the lowest value. This phenomenon was due to the difference in the water content of the materials which directly affects the Young’s modulus since water acts as a plasticizer [50]. For all starch nano-biocomposites, whatever the type of plasticizer, a reinforcement effect with an increase in the matrix stiffness was clearly observed with addition of halloysite. Such enhancement in modulus had been widely reported in the literature for other starch-based nanocomposites systems [40,50]. This has been attributed to (i) the addition of the halloysite nanoclay which has a high elastic modulus of about 140 GPa [25], and (ii) the interactions between the nanofillers and the plasticized starch matrix. Besides, the new crystalline structure induced from halloysite addition led to strong interactions which contributed to the stiffness increase through improved load transfer between the matrix and the nanofiller. The theoretical Young’s moduli of nanocomposites with polyols mixture were calculated according to Equation (2):

Y_mixture_ = Y_glycerol_ V_glycerol_ + Y_sorbitol_ V_sorbitol_ (2)

where Y_glycerol_ is the Young’s modulus of the glycerol-plasticized starch nanocomposite, Y_sorbitol_ is the Young’s modulus of the sorbitol-plasticized starch nanocomposite, V_glycerol_ and V_sorbitol_ are the corresponding nanocomposite volume fractions in the systems. The ratio of V_glycerol_/V_sorbitol_ is 0.6/0.4. Since the Young’s moduli of nanocomposites with sorbitol as plasticizer were much higher than those of nanocomposites with glycerol, the theoretical values were mainly dependent on sorbitol nanocomposites. The calculated values for P0, P3, P5 and P7 were 115.6, 127.6, 133.5 and 139.0 MPa, respectively. These values were lower than the experimental ones. This was probably due to (i) the much better dispersion state of nanofillers in the matrix based on the polyols mixture compared to that in the sorbitol-based matrix, and (ii) the lower experimental water content compared to the theoretical values [50].

Similar to Young’s modulus evolutions, the tensile strength of plasticized starch was significantly impacted by the nature of the plasticizer. The tensile strength of starch plasticized with the polyols mixture was higher than the glycerol plasticized one. The highest tensile strength value was obtained with sorbitol. Such a trend was also recently observed in plasticized alginate obtained by thermo-mechanical mixing [51]. This was due to the higher plasticizing efficiency of glycerol compared to sorbitol and to higher water uptake after equilibration with glycerol. Zhang and Han [52] studied the plasticizing effects of polyols on pea starch films and concluded that glycerol had higher plasticization efficiency due to the small size of the molecule, which can easily locate between the starch chains and disrupt intermolecular polymer interactions while sorbitol had larger sized molecules which can reduce its plasticizing effect. Compared to the neat matrix, whatever the type of plasticizer, an increase in the nanocomposites tensile strength was obtained and was correlated to the clay content. This enhancement seemed to be more pronounced for the glycerol plasticized nano-biocomposites, with an increase of up to 47% for the highest clay loading compared to a roughly 11% increase for the sorbitol and polyols mixture plasticized systems. This was also attributed to better nanofiller dispersion and stronger halloysite/matrix interactions leading to better load transfer between the main components during the uniaxial test. On the other hand, it should be noted that the nanocomposites based on both sorbitol and polyols mixture as plasticizers exhibited an insignificant increase in tensile strength, probably due to aggregation of halloysite at 5 wt.% and 7 wt.% loadings. The theoretical tensile strength of nanocomposites with polyols mixture were also calculated according to Equation (3):

T_mixture_ = T_glycerol_ V_glycerol_ + T_sorbitol_ V_sorbitol_, (3)

where T_glycerol_ is the tensile strength of the nanocomposite with glycerol-plasticized starch, T_sorbitol_ is the tensile strength of the nanocomposite with sorbitol-plasticized starch, V_glycerol_ and V_sorbitol_ are the corresponding nanocomposite volume fractions in the systems. The calculated values for P0, P3, P5 and P7 were 5.25, 5.68, 5.97 and 6.33 MPa, respectively. As expected, the experimental tensile strength values were higher than the theoretical ones.

The nature of the plasticizer also impacted the elongation at break values of neat plasticized matrices. The elongation at break of the glycerol plasticized starch was lower than the sorbitol ones (alone or in mixture) probably due to the nanofiller dispersion state in the matrix. According to the morphological analyses, nanocomposites based on glycerol displayed good halloysite dispersion while nanocomposites based on sorbitol and the polyols mixture as plasticizers showed some aggregation of nanofiller at a high degree of loading. These results were in agreement with some previous observations off plasticized starch stored at high RH [37,53]. Due to the higher hydrophilic character of glycerol compared to sorbitol, the overall content of plasticizer (water included) for materials based on glycerol was higher than that in materials based on sorbitol or in the polyols mixture (sorbitol and glycerol). In the presence of a high amount of water, the relatively strong hydrogen bonds between starch-polyol and starch-starch molecules are partially replaced by the weaker hydrogen bonds between starch-water and polyol-water, resulting in decreased deformation [37]. Besides, according to XRD analysis and compared to glycerol, sorbitol crystallized during storage, which decreased the amount of efficient plasticizing polyol in the starch matrix and could also have enhanced “crosslink” formation in the starch network and then impacted the elongation at break properties [37]. This behavior has also been observed in plasticized alginate with high plasticizer content [51,54]. The elongation properties of the nanocomposites showed clear and different trends depending on the plasticizer’s nature. The incorporation of halloysite nanoclay into the glycerol plasticized starch induced slightly increased elongation at break values related to the homogenous dispersion of nanofiller in the matrix and decreased water content. On the contrary, the values for polyols mixture and sorbitol-based systems decreased with increases in nanoclay content. Such behaviors were attributed to aggregates of halloysite nanoclay in the matrix, as observed by SEM, which embrittled the materials. Chivrac et al. [50] also confirmed that glycerol plasticized starch/montmorillonite (MMT) nanocomposites showed no variation in the strain at break with increasing clay contents because of high exfoliation and the good dispersion state of MMT in the starch matrix, while sorbitol plasticized nano-biocomposites showed slightly decreased extensibility due to remaining small clay tactoids which embrittled the plasticized starch matrices.

## 4. Conclusions

Plasticized starch/halloysite nano-biocomposites were successfully prepared. The influence of plasticizer type and filler loading on the microstructure and properties of the resulting materials were studied. Glycerol contributed to a more homogenous dispersion of halloysite nanotubes in the matrix compared to sorbitol, used alone or in combination, due to stronger and more stable hydrogen bonds between glycerol plasticized starch and halloysite, as revealed by ATR-FTIR analyses. The XRD results showed that incorporation of halloysite nanoclay led to the formation of a new crystalline structure attributed to amylopectin crystallization at the nanofiller interface which was mainly observed for nanocomposites based on glycerol or the mixture of glycerol and sorbitol as plasticizers. Whatever the type of plasticizer, the incorporation of halloysite increased the thermal stability and reduced the moisture content of the starch nano-biocomposites. The mechanical properties were also greatly impacted by the nature of the plasticizer. Glycerol-plasticized starch exhibited lower Young’s modulus, tensile strength and elongation at break than the sorbitol one. After addition of halloysite nanoclay, the improvement in tensile properties was more pronounced for glycerol plasticized systems due to the better dispersion state of halloysite compared to the systems based on sorbitol-plasticized starch. Finally, the use of mixtures of these polyols was found to be a good and promising way to optimize the mechanical properties of these starch-based nanocomposites, since the values of Young’s modulus and tensile strength were in-between those of glycerol- and sorbitol-based nanocomposites and higher than the theoretically expected ones.

This work clearly showed that halloysite nanoclay is an effective and promising clay for enhancing the properties of plasticized starch. Nano-biocomposites based on starch/halloysite nanoclay represent an interesting alternative to replace some non-biodegradable materials in different fields such as packaging, agriculture or biomedical applications. For instance, in active packaging or in specific biomedical applications, halloysite nanoclay could be a good carrier for some active drugs due to its specific structure and surface properties. In future works, we will focus on developing different functional biomaterials based on starch/halloysite nanocomposites in order to broaden the properties and applications of starch-based materials.

## Figures and Tables

**Table 1:** TGA data for the starch-based nanocomposites and neat glycerol, sorbitol and halloysite.

Sample Code	T_10%_ (°C)	T_50%_ (°C)	T_90%_ (°C)	T_max_ (°C)	Char Residue (%)
G0	131	291	337	287	0
G3	161	293	373	289	2.1
G5	195	295	463	291	4.9
G7	194	298	511	306	7.0
P0	189	297	361	288	0
P3	230	301	401	288	3.1
P5	226	300	429	290	4.6
P7	232	300	496	304	7.1
S0	223	297	349	297	0
S3	239	300	401	298	2.2
S5	248	300	448	299	4.7
S7	245	300	467	306	6.3
Glycerol	127	154	162		-
Sorbitol	218	251	268		-
Halloysite	405	>600	>600		-

**Table 2:** Mechanical properties, moisture content and densities of the different samples.

Sample Code	Stress at Break (MPa)	Strain at Break (%)	Young’s Modulus (MPa)	Moisture Content (%)	Density (g/cm^3^)
G0	2.28 ± 0.13	26.1 ± 2.3	22.0 ± 2.2	16.7 ± 0.2	1.45 ± 0.02
G3	2.64 ± 0.11	27.3 ± 2.6	28.3 ± 1.9	15.0 ± 0.2	1.47 ± 0.01
G5	2.94 ± 0.05	29.3 ± 2.3	34.6 ± 2.2	14.2 ± 0.2	1.48 ± 0.01
G7	3.36 ± 0.24	34.5 ± 3.6	37.0 ± 2.7	13.8 ± 0.2	1.50 ± 0.01
P0	7.13 ± 0.06	42.4 ± 1.7	119.2 ± 4.9	11.1 ± 0.1	1.48 ± 0.01
P3	7.36 ± 0.22	39.6 ± 1.9	144.7 ± 12.0	10.6 ± 0.1	1.49 ± 0.01
P5	7.77 ± 0.12	37.0 ± 0.4	194.1 ± 15.3	10.4 ± 0.1	1.50 ± 0.01
P7	7.88 ± 0.22	33.2 ± 3.0	174.5 ± 8.1	10.0 ± 0.1	1.52 ± 0.01
S0	9.70 ± 0.79	43.3 ± 3.0	256.0 ± 19.0	9.6 ± 0.1	1.49 ± 0.03
S3	10.24 ± 0.48	39.1 ± 2.7	276.5 ± 28.8	8.4 ± 0.1	1.50 ± 0.02
S5	10.52 ± 0.51	36.5 ± 5.4	281.8 ± 19.0	8.7 ± 0.1	1.52 ± 0.01
S7	10.78 ± 0.37	35.0 ± 5.6	292.1 ± 34.9	7.5 ± 0.1	1.54 ± 0.02
